# Supplementary material for: ODT FLOW: Extracting, analyzing, and sharing multi-source multi-scale human mobility
Source: PLoS One. 2021 Aug 5;16(8):e0255259. doi: 10.1371/journal.pone.0255259 (PMC8341631; doi:10.1371/journal.pone.0255259)
Supplement: S1 File — (PDF) [file pone.0255259.s001.pdf]

S1 File

The derived multi-source multi-scale human mobility data are open sourced and can be accessed via the interactive ODT Flow Explorer at http://gis.cas.sc.edu/GeoAnalytics/od.html. A video tutorial showing how to use the ODT Flow Explorer is available at https://www.youtube.com/watch?v=lV3AJIVYnSI.

Tutorial (code examples) of how to access the ODT flow data programmatically using the ODT Flow REST APIs with Jupyter Notebook is available at https://github.com/GIBDUSC/ODT_Flows/blob/main/ODT%20Flow%20REST%20APIs_Notebook_Tutorial.ipynb

A copy of all the source code of the case studies including the Jupyter Notebooks and KNIME workflows is available at Harvard Dataverse (https://doi.org/10.7910/DVN/GL3HAB).
